# Epinephrine Stimulates *Mycobacterium tuberculosis* Growth and Biofilm Formation

**DOI:** 10.3390/ijms242417370

**Published:** 2023-12-12

**Authors:** Yingying Lei, Khaista Rahman, Xiaojian Cao, Bing Yang, Wei Zhou, Aikebaier Reheman, Luxia Cai, Yifan Wang, Rohit Tyagi, Zhe Wang, Xi Chen, Gang Cao

**Affiliations:** 1 National Key Laboratory of Agricultural Microbiology, College of Veterinary Medicine, Huazhong Agricultural University, Wuhan 430070, China; leiyingying@webmail.hzau.edu.cn (Y.L.); khaistaqau@gmail.com (K.R.); Stargazer@webmail.hzau.edu.cn (X.C.); ybing1992@163.com (B.Y.); zhouwei0715@webmail.hzau.edu.cn (W.Z.); akpar0902@163.com (A.R.);; 2 Shanghai Collaborative Innovation Center of Agri-Seeds/School of Agriculture and Biology, Shanghai Jiao Tong University, Shanghai 200240, China; 3 Bio-Medical Center, Huazhong Agricultural University, Wuhan 430070, China

**Keywords:** *M. tuberculosis*, *M. smegmatis*, epinephrine, *mprB*, biofilm, antibiotic resistance

## Abstract

The human stress hormones catecholamines play a critical role in communication between human microbiota and their hosts and influence the outcomes of bacterial infections. However, it is unclear how *M. tuberculosis* senses and responds to certain types of human stress hormones. In this study, we screened several human catecholamine stress hormones (epinephrine, norepinephrine, and dopamine) for their effects on *Mycobacterium* growth. Our results showed that epinephrine significantly stimulated the growth of *M. tuberculosis* in the serum-based medium as well as macrophages. In silico analysis and molecular docking suggested that the extra-cytoplasmic domain of the MprB might be the putative adrenergic sensor. Furthermore, we showed that epinephrine significantly enhances *M. tuberculosis* biofilm formation, which has distinct texture composition, antibiotic resistance, and stress tolerance. Together, our data revealed the effect and mechanism of epinephrine on the growth and biofilm formation of *M. tuberculosis*, which contributes to the understanding of the environmental perception and antibiotic resistance of *M. tuberculosis* and provides important clues for the understanding of bacterial pathogenesis and the development of novel antibacterial therapeutics.

## 1. Introduction

Tuberculosis (TB), caused by *Mycobacterium tuberculosis* (*M. tuberculosis*), is the second leading infectious disease after COVID-19. The emergence of drug-resistant TB, especially multidrug-resistant TB (MDR-TB) and extensively drug-resistant TB (XDR-TB), is considered the greatest obstacle to global TB control. People infected with *M. tuberculosis* have a 5–10% lifetime risk of falling ill with TB. Those with compromised immune systems, such as people living with HIV and malnutrition, have a higher risk of falling ill [1].

Stress impairs major immune functions through multiple pathways, consequently affecting the secretion of the neuroendocrine hormones glucocorticoids and catecholamines [2]. Stress-induced catecholamines, epinephrine (Epi), norepinephrine (NE), and dopamine (Dop), affect the homeostasis of the body and influence the course of numerous diseases [3]. It has also been demonstrated that stress-induced catecholamines influence bacterial growth and virulence, thereby affecting their interaction with the host and the outcomes of bacterial infections [4,5,6]. The interaction of gut bacteria with stress hormones can stimulate the growth, motility, virulence, and/or biofilm formation of pathogens such as *Escherichia coli*, *Salmonella* spp., *Salmonella Enteritidis*, and *Yersinia ruckeri* [7,8,9,10].

When residing within host niches, bacteria could employ an array of molecular sensors to adapt to their environment changes [11,12]. While invading the host, bacteria sense these hormones through catecholamine receptors [5]. As an intracellular bacterium, *M. tuberculosis* encounters various chemical signals including catecholamines. Previous studies have shown the effects of some hormones on the pathogenicity and growth of *M. tuberculosis* inside an infected host. For example, sexual hormones substantially modify the host immune system activity and influence the course of experimental pulmonary TB [13]. Endocrine hormones were found to modify the cellular immune responses of TB patients [14]. Cortisol and/or dehydroepiandrosterone (DHEA) modify the immunomodulatory capability and intracellular bacterial growth of THP-1-derived macrophages infected with *M. tuberculosis* [15]. However, the effect of catecholamines on *M. tuberculosis* and the underlying mechanism remain elusive.

In the current study, we investigated the effect of catecholamines on the growth, biofilm formation, and antibiotic and stress tolerance of *M. tuberculosis* and deciphered the underlying mechanism.

## 2. Results

### 2.1. Epinephrine Stimulates M. tuberculosis Growth In Vitro

**Figure 1:**
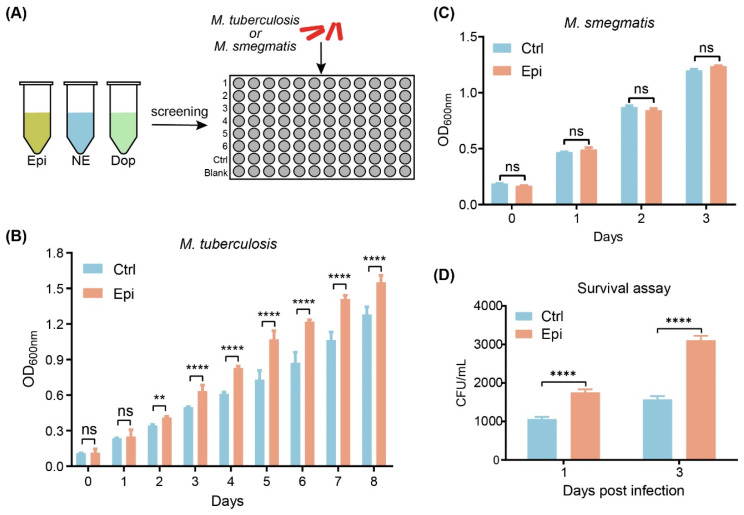
Epi stimulates *M. tuberculosis* H37Ra growth in vitro. (**A**) Flow chart of the effect of catecholamine stress hormones on the growth of *M. tuberculosis* and *M. smegmatis*. (**B**) Effects of Epi on the growth of *M. tuberculosis* on serum-7H9 medium. (**C**) Effects of Epi on the growth of *M. smegmatis* on serum-7H9 medium. (**D**) Effects of Epi on *M. tuberculosis* CFU in THP-1 cells. PMA-primed THP-1 cells were pretreated with 2 μM Epi or vehicle control for 24 h, challenged with *M. tuberculosis* (MOI = 3) for 6 h, and then treated with DMSO or epinephrine for 12 h and 3 days, respectively. The intracellular viable bacilli were determined by CFU at the indicated time. ****, *p* < 0.0001; **, *p* < 0.01; ns, not significant (two-way ANOVA). Data are representative of three independent experiments with three biological replicates (mean ± SEM).

Catecholamine stress hormones can significantly influence the growth and behavior of various bacteria [9,16,17,18]. To investigate the effect of catecholamines on the growth of *M. tuberculosis* or *M. smegmatis*, we incubated *M. tuberculosis* or *M. smegmatis* with epinephrine (Epi), norepinephrine (NE), or dopamine (Dop) and examined the OD_600nm_ (Figure 1A). The results showed that the in vitro growth of *M. tuberculosis* was significantly increased in the presence of Epi, but not in the presence of NE and Dop, as compared to the vehicle treatment (Figure 1B and Figure S1A,B). Of note, none of these three hormones showed significant effects on the growth of *M. smegmatis* in vitro (Figure 1C and Figure S1C).

Next, we tested the effect of Epi on intracellular *M. tuberculosis* growth by performing a survival assay. We observed that the viable *M. tuberculosis* was significantly increased in Epi-treated macrophages compared to the vehicle control (Figure 1D and Figure S1D). Cell viability was measured by MTS, which showed a significant difference between vehicle and Epi treatment (Figure S1D). These data indicated that Epi has a positive effect on the growth of *M. tuberculosis* in vitro and in macrophages.

### 2.2. MprB Is Involved in the Regulation of M. tuberculosis Growth In Vitro by Epi

**Figure 2:**
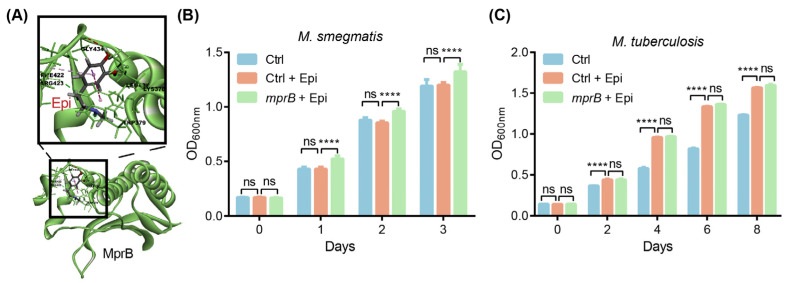
MprB is involved in regulation of *M. tuberculosis* growth in vitro by Epi. (**A**) Docking of Epi to *M. tuberculosis* MprB. (**B**) Effect of 2 μM of Epi on the growth of *M. smegmatis* overexpressing *mprB*. (**C**) Effects of 2 μM of Epi on the growth of *M. tuberculosis* overexpressing *mprB*. ****, *p* < 0.0001, ns, not significant (two-way ANOVA). Data are representative of three independent experiments with three biological replicates (mean ± SD).

MprAB two-component systems (TCSs) are involved in sensing external environmental signals and controlling stress response in mycobacterial species [19,20]. We hypothesized that *M. tuberculosis* equips some proteins to sense Epi. Molecular docking analyses were performed to predict the binding sides between MprB and Epi. Our results show that MprB is able to bind to Epi (Figure 2A), suggesting that MprB might be the potential sensor of Epi. Further, we overexpressed *mprB* in *M. tuberculosis* and *M. smegmatis* mc^2^ 155, respectively, and examined the growth of bacteria with or without Epi exposure in vitro. Our results showed that the overexpression of *mprB* significantly enhanced the growth of *M. smegmatis* but not that of *M. tuberculosis* with the treatment of Epi in comparison to the wild-type strain (Figure 2B,C). We then knocked down *mprB* from *M. tuberculosis* (*mprB^KD^*) and found that the Epi-treated *mprB^KD^* strain reduced the growth of *M. tuberculosis* compared to the Epi-treated wild-type strain (Figures S1 and S2). These results suggest that *M. tuberculosis mprB* might be the putative adrenergic sensor.

### 2.3. Epi Stimulates M. tuberculosis Biofilm Formation

**Figure 3:**
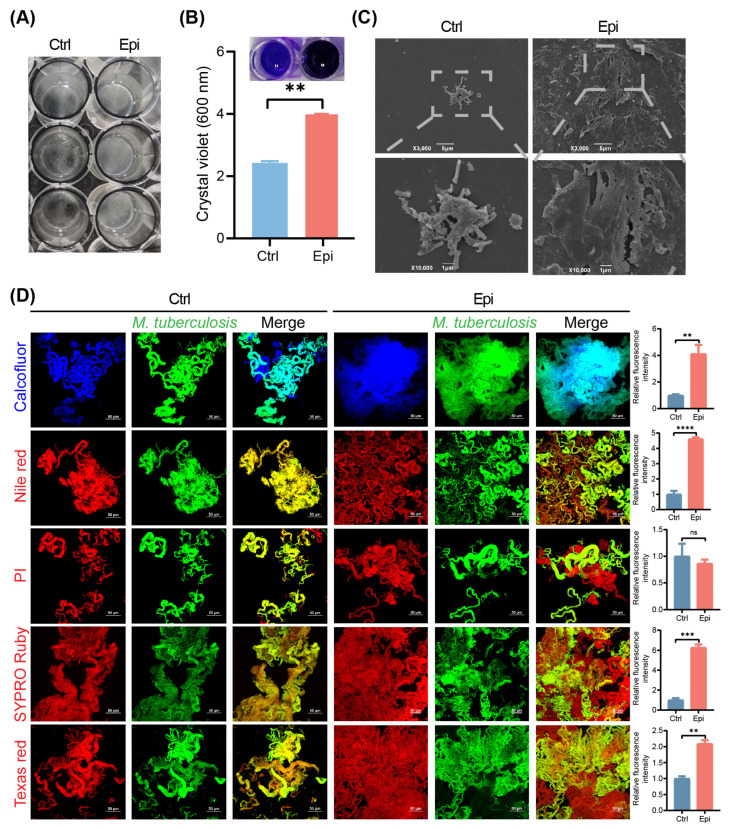
Epi affects *M. tuberculosis* biofilm. (**A**) Exponential cultures of *M. tuberculosis* were exposed to 2 μM of Epi for 1 week. (**B**) CV assays were performed to quantitate *M. tuberculosis* biofilms. The data are represented as mean ± SD. Statistical significance was determined using Student’s *t* test. **, *p* < 0.01. (**C**) *M. tuberculosis* biofilms were developed in the serum-7H9 medium. SEM images are shown at ×3000 (**upper**) and ×10,000 (**bottom**). (**D**) Characterization of Epi-induced biofilm matrices in *M. tuberculosis*. *M. tuberculosis* carrying eGFP were subjected to 2 μM of Epi for 1 week and then stained with Calcofluor white (for carbohydrates), Nile red (for lipids), PI (for eDNA), SYPRO Ruby (for proteins), and Texas red (for polysaccharides), respectively. Cultures were then analyzed using CLSM. All data are representative of three independent biological experiments performed in triplicate. Scale bars in (**D**) indicate 50 µm. The data are represented as mean ± SEM. Statistical significance was determined using Student’s *t* test. ****, *p* < 0.0001; ***, *p* < 0.001; **, *p* < 0.01; ns, not significant.

Catecholamine hormones are reported to enhance biofilm formation [21] and antibiotic resistivity in some bacteria [22]. Thus, we investigated the role of Epi on the biofilm formation of *M. tuberculosis*. Our data showed that when exposed to Epi, *M. tuberculosis* grew faster than the vehicle-treated bacteria and significantly increased the absorbance of crystal violet at OD_600nm_ (Figure 3A,B), indicating that Epi stimulates *M. tuberculosis* biofilm formation. Next, the biofilms were analyzed under a scanning electron microscope (SEM), which revealed a bunch of bacteria embedded in biofilms with the treatment of Epi. As shown in Figure 3C, the Epi-induced biofilm was more compact and smoother than the vehicle-treated bacteria.

The hallmark of biofilm is the self-production of the extracellular polymeric substance (EPS), mainly composed of exopolysaccharides, lipids, secreted proteins, and extracellular DNAs [23]. To further analyze the biochemical compositions of the Epi-induced *M. tuberculosis* biofilm, we stained the biofilm with Calcofluor white, Nile red, propidium iodide (PI), SYPRO Ruby, and Texas Red for carbohydrates, lipids, extracellular DNA, proteins, and polysaccharides, respectively. As shown in Figure 3D, more carbohydrates, lipids, proteins, and polysaccharides were observed in the Epi-induced biofilm than in the vehicle control bacteria. These data demonstrated that Epi-induced *M. tuberculosis* biofilm comprises textures and compositions.

### 2.4. Epi Enhances M. tuberculosis Antibiotic Resistivity and Stress Tolerance

**Figure 4:**
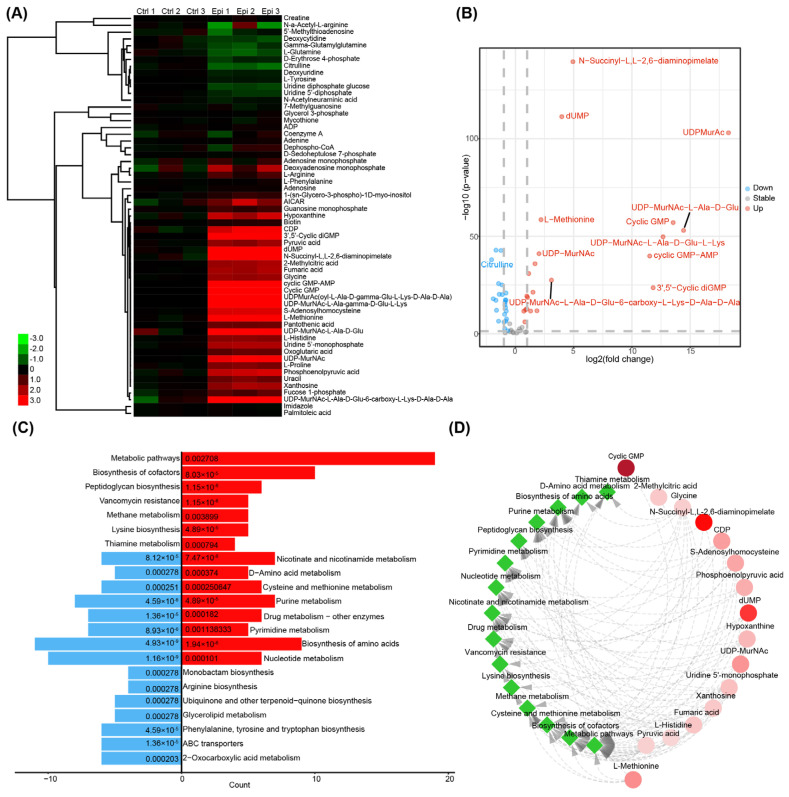
Different metabolome between Epi-treated and vehicle-treated *M. tuberculosis*. (**A**) Heat map and clustering presenting metabolomic profiles of Epi-treated and vehicle-treated *M. tuberculosis*. (**B**) Volcano plot indicating significant up-regulated (red) and down-regulated (blue) metabolites from LC-MS-based metabolomics data. Red and blue dots indicate metabolites with higher abundance in Epi-treated and vehicle-treated bacteria, respectively. (**C**) KEGG enrichment of metabolites that had significant change between Epi-treated and vehicle-treated *M. tuberculosis*. The red bar indicates metabolites with higher abundance in Epi-treated bacteria-enriched pathways and the blue bar indicates metabolites with higher abundance in vehicle-treated bacteria-enriched pathways. *p* values of each enriched pathway were marked at the end of the bar, while the length of the bar indicated the number of enriched metabolites in each pathway. (**D**) The network between up-regulated metabolites with KEGG pathways. Each green node represented a pathway and each circle edge indicated enriched metabolites; foldchange (Epi-treated vs. vehicle-treated) was illustrated with the color of the circle node, the edge between the nodes indicating the belonging of metabolites to the pathway.

To further investigate the difference in the biochemical contents between *M. tuberculosis* treated with/without Epi, we performed metabolite analyses. Our data revealed 59 metabolites with significant differences between the control and *M. tuberculosis* exposed to Epi (Figure 4A and Table S1), of which 29 metabolites showed higher abundance in Epi-treated bacteria (Figure 4B). KEGG enrichment demonstrated that the different metabolites mainly consisted of DNA, RNA, and amino acid metabolism (Figure 4C). Of note, 3′,5′-Cyclic diGMP (cGMP) (KEGG C16463) participating in the two-component system and biofilm formation, Cyclic GMP (KEGG C00942) participating in purine metabolism, cyclic GMP-AMP (KEGG C20640) participating in the cytosolic DNA-sensing pathway, and UDPMurAc(oyl-L-Ala-D-gamma-Glu-L-Lys-D-Ala-D-Ala) (KEGG R05629), UDP-MurNAc-L-Ala-gamma-D-Glu-L-Lys (KEGG C05892), UDP-MurNAc-L-Ala-D-Glu (KEGG C00692), UDP-MurNAc, UDP-MurNAc-L-Ala-D-Glu-6-carboxy-L-Lys-D-Ala-D-Ala (KEGG C04882), and UDP-MurNAc (KEGG C01050) participating in peptidoglycan biosynthesis were significantly more abundant in the Epi-treated *M. tuberculosis* (Figure 4D).

**Figure 5:**
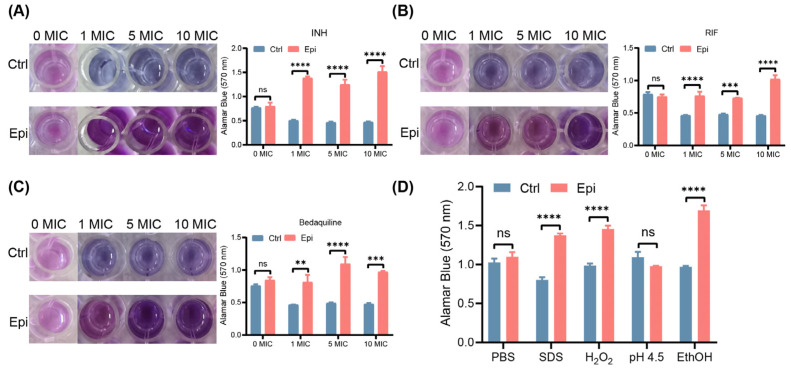
Epi enhances *M. tuberculosis* antibiotic resistivity and stress tolerance. (**A**–**C**) *M. tuberculosis* biofilms were treated with INH (**A**), RIF (**B**), and Bedaquiline (**C**) at 0×, 1×, 5×, and 10× MIC for 72 h, respectively, and then assayed by drug susceptibility analysis. (**D**) Effects of Epi on the in vitro *M. tuberculosis* stress tolerance. The data are represented as mean ± SEM. Statistical significance was determined using two-way ANOVA. ns, not significant; **, *p* < 0.01; ***, *p* < 0.001; ****, *p* < 0.0001. All data are representative of three independent biological experiments performed in triplicate.

Peptidoglycan biosynthesis is organized into networks with varying drug susceptibility [24]. Moreover, the metabolites involved in the biosynthesis of cofactors and vancomycin resistance are highly abundant in Epi-treated bacteria. As these metabolites are associated with bacterial drug resistance and biofilm formation [25], we then performed drug susceptibility analysis with the treatment of different concentrations of drugs. Our data demonstrated that the OD_570nm_ of Epi-treated *M. tuberculosis* was significantly increased compared to the control bacteria when incubated with INH, RIF, and Bedaquiline (Figure 5A–C), indicating the Epi-induced biofilm formation facilitates *M. tuberculosis* drug tolerance. Furthermore, we investigated the role of Epi-induced biofilm formation in stress tolerance, such as 0.1% SDS, 10 mM H_2_O_2_, low pH (pH 4.5), and 5% ethanol. Our data showed that the OD_570nm_ of Epi-treated *M. tuberculosis* was significantly higher than that of the vehicle control when incubated with SDS, H_2_O_2_, and ethanol, but not pH 4.5 (Figure 5D). Together, these findings indicated that Epi induces antibiotic and stress tolerance in *M. tuberculosis*.

## 3. Discussion

The disturbance of hormonal homeostasis occurs during TB and has a significant impact on the host’s immune system. The elevated level of stress hormones negatively regulates the immune system and increases the risk of infection [2]. Despite disturbing the immune system, stress hormones also influence the pathogenicity and survival of microbes inside the host [26,27,28]. In the current study, we proved for the first time that catecholamine hormones such as Epi affected the growth of *M. tuberculosis* in both serum-based media and macrophages (Figure 1). In-silico analysis suggested that MprB may be responsible for sensing catecholamines. This hypothesis was supported by the overexpressing *mprB* in *M. smegmatis*, leading to increased responsiveness to Epi. However, we did not find any significant difference between the wild-type and *mprB* overexpressed *M. tuberculosis* strains upon treatment with the Epi (Figure 2). We speculate that this may be due to the hysteresis nature of the MprAB system in *M. tuberculosis* [29]. We tried to knock out *mprB* from *M. tuberculosis* but failed due to its growth essentiality. We then tried to knock down *mprB* from *M. tuberculosis* (*mprB^KD^*) and examined whether Epi promotes *M. tuberculosis* proliferation through *mprB*. The Epi-treated *mprB^KD^* strain reduced the growth of *M. tuberculosis* compared to the wild-type strain, but Epi still promoted the growth of the *mprB^KD^* strain on day 6 and day 8. The possible reason is that the function of *mprB* is compensated by other genes or the poor knockdown effect (~20%, Figure S2). Therefore, it would be important to further confirm the adrenergic sensors for catecholamines and underlying mechanisms.

We found that Epi can also affect the extracellular matrix of *M. tuberculosis*, which was validated by biofilm CV assay and SEM (Figure 3). Our metabolome data suggested distinct metabolites spectra between *M. tuberculosis* treated with/without Epi: 3′,5′-Cyclic diGMP were found to have significantly higher abundance in the Epi-treated bacteria (Figure 4A,B); 3′,5′-Cyclic diGMP could activate the production of adhesins and extracellular matrix products, leading to the formation of biofilm in *P. aeruginosa* [30] and *V. cholerae* [31]. This suggested that Epi treatment might induce the production of 3′,5′-Cyclic diGMP, which increases the biofilm formation of *M. tuberculosis*. Mycobacterial biofilm mainly contains mycolic acids (lipids), proteins, eDNA, and polysaccharides [32,33]. The composition of the biofilms affects the texture and stability of biofilms [34]. Thus, we applied different dyes to stain different constituents of the biofilm and found more carbohydrates, lipids, proteins, and polysaccharides in the Epi-induced biofilm using CLSM, which was consistent with our metabolite analysis (Figure 3D and Figure 4). It has been demonstrated that biofilm protects bacteria against environmental stress such as cell envelope integrity [35], oxidation [36], and antibiotic resistance [37,38]. The increased polysaccharides and lipids may be responsible for producing the drug tolerance phenotype in Epi-treated bacteria. In line with this, we found that Epi-treated bacteria were more resistant to antibiotics, SDS, H_2_O_2_, and ethanol (Figure 5), of which the mechanisms need to be further investigated. Moreover, we observed this phenomenon in attenuated the *M. tuberculosis* H37Ra strain, and this phenomenon may be different in different strains [39]. Our data suggest that the application of quorum-sensing inhibitors to increase biofilm susceptibility to antibiotics may be a potential strategy to address Epi-induced *M. tuberculosis* biofilm formation.

In conclusion, our data showed that catecholamine Epi enhances *M. tuberculosis* growth in serum serum-based media and affects the extracellular matrix of *M. tuberculosis*. Further work needs to be conducted to elucidate the effect of catecholamines, especially Epi, on *M. tuberculosis* in a suitable animal model and the detailed mechanisms. Together, our data contribute to the understanding of the environmental perception and antibiotic resistance of *M. tuberculosis*.

## 4. Materials and Methods

### 4.1. Bacteria Strains, Growth Conditions, and Reagents

*Mycobacterium tuberculosis* (*M. tuberculosis*) H37Ra and *Mycobacterium smegmatis* (*M. smegmatis*) mc^2^ 155 were cultured at 37 °C under static conditions in Middlebrook 7H9 broth (Becton Dickinson, Bergen County, NJ, USA, 271310) supplemented with 10% oleic albumin dextrose catalase (OADC, Becton Dickinson, NJ, USA), 0.05% Tween 80, and 0.5% glycerol or on solid Middlebrook 7H11 agar plates (Becton Dickinson, NJ, USA) supplemented with 10% OADC and 0.5% glycerol. When required, a final concentration of 25 μg/mL kanamycin was added to the medium.

For checking the effects of hormones on the growth of *M. tuberculosis* or *M. smegmatis*, bacteria were treated according to the procedure described previously [40]. Briefly, bacterial cultures from the logarithmic phase were sub-cultured in Middlebrook 7H9 broth supplemented with 10% OADC, 0.5% glycerol, 10% fetal bovine serum (FBS), and 10 nmol/L ascorbic acid (serum-7H9) and the OD_600nm_ was adjusted to ∼0.1. The bacterial culture was left untreated (blank control) or exposed to 2 μM of epinephrine (Epi, Sigma, Darmstadt, Germany, E4642), norepinephrine (NE, Aladdin, Shanghai, China, N107258), or dopamine (Dop, Sigma, Darmstadt, Germany, H8502), respectively. DMSO-treated bacteria were used as a control.

For the in vitro bacterial growth assay, bacteria were cultured in T25 polystyrene flasks with serum-7H9 broth and the initial OD_600nm_ was adjusted to ∼0.1. For *M. tuberculosis* H37Ra, bacterial OD_600nm_ was measured daily for 8 days and CFU was measured at day 0, day 4, and day 8. For *M. smegmatis* mc^2^ 155, bacterial OD_600nm_ was measured daily for 3 days. All samples and experiments were performed in triplicate.

### 4.2. Colony-Forming Unit (CFU) Assay

The human monocytic leukemia cell line THP-1 (ATCC, TIB-202) was cultured in a RPMI-1640 medium supplemented with 10% FBS, 100 U/mL penicillin, and 100 μg/mL streptomycin at 37 °C in 5% CO_2_. Then, 10^5^ cells per well were seeded in 24-well plates and were differentiated for 24 h by supplementing 40 ng/mL phorbol 12-myristate 13-acetate (PMA) at 37 °C in 5% CO_2_. The cells were infected with *M. tuberculosis* H37Ra (MOI = 3) for 6 h and then washed thrice with pre-warmed PBS and supplied with a fresh medium with 5% FBS containing amikacin (50 µg/mL) for 1 h to eliminate the extracellular bacteria (referred to as day 0). Cells were then incubated with the fresh RPMI-1640 medium in the presence or absence of 2 μM of Epi. The medium was changed every 12 h. The infected cells were lysed at indicated time points using 0.5 mL of sterile 0.1% Tween 80 in water, and viable *M. tuberculosis* was enumerated by 10-fold serial dilution of lysates and plating in triplicate over 7H11 agar plates. Plates were incubated for 3 weeks and colonies were quantified.

### 4.3. Construction of the mprB Overexpressing Strain of M. tuberculosis H37Ra and M. smegmatis mc^2^ 155

*mprB* (Genebank NC_000962.3) was amplified using primers *mprB*-F (5′-CAGAATTCATGTGGTGGTTCCGCCGCCG-3′) and *mprB*-R (5′-CTCAGTCCACGCGCGCAACCTAGAGATCTCG-3′) and cloned into vector pMV261-psmyc. The plasmid was electro-transformed into *M. tuberculosis* H37Ra or *M. smegmatis* mc^2^ 155 and cultured on 7H11 agar plates containing 25 μg/mL kanamycin. The empty pMV261-psmyc vector was used as the vector control.

### 4.4. Construction of the mprB Knock down Strain of M. tuberculosis H37Ra

The *mprB* gene was knocked down in *M. tuberculosis* H37Ra by the Cas 10 RNA interference method [41]. The downregulation of *mprB* was confirmed by qRT-PCR using primers qPCR-*mprB*-F (GTGATCCGTGGCGAGTTGTTCAT) and qPCR-*mprB*-R (TGCTTCGGTGGGCTTGAGACTT) (Figure S2A).

### 4.5. Molecular Docking

Discovery Studio 3.1 (Accelrys Co., Ltd., San Diego, CA, USA) access was provided by the Huazhong Agricultural University (Wuhan, China). The *M. tuberculosis* Rv0982 MprB (PDB code: 6BLK) crystal structures were downloaded from RCSB Protein Data Bank (https://www.rcsb.org) (accessed on 11 June 2020). Hybridization states, charges, and angles were assigned in the protein structure with missing bond orders and explicit hydrogen atoms were added at pH 7.4. The energy of the protein structure was minimized in 200 steps of the smart minimize method. To prepare ligands, the 3D structures of epinephrine (CID: 5816) were downloaded from the PubChem database (https://pubchem.ncbi.nlm.nih.gov) (accessed on 6 November 2020) and optimized with Discovery Studio 3.1. LigandFit and CDOCKER Docking programs implemented in Discovery Studio 3.1 were conducted following the study by Li et al. [42].

### 4.6. Crystal Violet (CV) Assay of Biofilm

The CV assay of *M. tuberculosis* H37Ra biofilm was performed as described previously [43]. Briefly, the CV assay was performed in 24-well plates under static conditions. Logarithmic-phase cultures of *M. tuberculosis* H37Ra (OD_600nm_ ~1) were diluted 1:100 in a serum-7H9 medium with or without 2 μM Epi. The plates were wrapped twice in parafilm and incubated at 37 °C for about 1 week. After biofilm formation, the medium was removed from wells by pipetting underneath the biofilm. Biofilms were dried in a biosafety cabinet and incubated with 500 μL of 1% CV for 10 min at 37 °C. The CV was removed and the M. tuberculosis H37Ra biofilm was gently washed twice with PBS. The bound CV was then extracted by a 10 min incubation with 1 mL of 95% ethanol at 37 °C. The absorption of extracted CV was measured at A600 on a spectrophotometer in a 96-well plate.

### 4.7. Scanning Electron Microscopy (SEM)

The cell morphology of *M. tuberculosis* H37Ra biofilms present on Epi treatment was assessed by SEM. A total of 300 μL from each sample of Epi and DMSO pretreated bacilli cultures were seeded in 48-well plates on sterilized glass coverslips. The plates were incubated at 37 °C for about 1 week. Then, the media were removed from each well and the coverslips were gently washed twice with pre-warmed PBS. The coverslips were transferred to a 2% glutaraldehyde solution and processed according to the previous study [44]. The slides were dried with 40% ethanol followed by 60%, 80%, and 100%, put in a dry oven at 37 °C for 1 h, and then put on clean aluminum pins and immobilized with Leit-C (Sigma, Darmstadt, Germany, 09929-30G). The pins were sputtered with Au (Agar Sputter Coater, Agar Scientific Ltd., Stansted, GB, UK) and subjected to scanning electron microscopy (SEM, JSM-6010LV, JEOL GmbH, Freising, Germany).

### 4.8. Confocal Laser Scanning Microscopy (CLSM)

*M. tuberculosis* H37Ra biofilms were produced on coverslips in a 24-well polystyrene plate using the methods described above. Biofilms were stained with fluorescent dyes such as 0.5 mg/mL Texas Red™ (ThermoFisher Scientific, Waltham, MA, USA, T1395MP), 1 mM Nile Red™ (ThermoFisher Scientific, Waltham, MA, USA, N1142), FilmTracer™ SYPRO™ Ruby Biofilm Matrix Stain (ThermoFisher Scientific, Waltham, MA, USA, F10318), 3 μg/mL Calcofluor white (ThermoFisher Scientific, Waltham, MA, USA, R40015), and 15 μM propidium iodide (PI, ThermoFisher Scientific, Waltham, MA, USA, P1304MP). Biofilms were stained with Texas Red, Nile Red, or SYPRO Ruby for 20 min with Calcofluor white for 30 min or with PI for 5 min. After staining, samples were washed three times with PBS and viewed using a Nikon confocal microscope.

### 4.9. Metabolite Extraction

*M. tuberculosis* was cultured in T25 polystyrene flasks with or without Epi, as shown above. After biofilm generation, both the control and Epi-treated cultures were centrifuged. The pellet masses were resuspended in 1 mL of a precooled mixture of acetonitrile/methanol/H_2_O (40:40:20). The suspensions were transferred to screw-headed 1.5 mL tubes and mechanical lysis with 0.1 mm zirconia beads in a Precellys tissue homogenizer for 3 min (6500 rpm) twice under continuous cooling at or below 2 °C. Lysates were clarified by centrifugation and then filtered across a 0.22 μm filter. The residual protein/peptide content of metabolite extracts (BCA Protein Assay kit; Thermo Scientific) was determined to normalize samples to cell biomass. All data obtained by metabolomics were averages of independent triplicates.

### 4.10. Metabolism Data Analysis and Visualization

Liquid chromatography–mass spectrometry (LC–MS)-based metabolomics was conducted according to previous literature [45,46]. Extracted metabolites were separated on a Cogent Diamond Hydride type C column (gradient 3) and the mobile phase consisted of solvent A (ddH_2_O with 0.2% formic acid) and solvent B (acetonitrile with 0.2% formic acid). The mass spectrometer used was an Agilent Accurate Mass 6220 time of flight (TOF) coupled to an Agilent 1200 liquid chromatography system. Detected ions were deemed metabolites based on unique accurate mass-retention time identifiers for masses exhibiting the expected distribution of accompanying isotopologues. Metabolite identities were searched using a mass tolerance of <0.005 Da. The relative concentration of metabolites was determined by using a calibration curve generated with varying concentrations of chemical standard spiked into a homologous mycobacterial extract to correct for matrix-associated ion suppression effects. The abundance of extracted metabolite ion intensities was extracted using Profinder 8.0 (Agilent Technologies, Santa Clara, CA, USA) and Qualitative Analysis 6.0 (Agilent Technologies) and normalized by each sample’s protein concentration. The clustered heat map and hierarchical clustering trees were generated using Cluster 3.0 (Stanford University, Stanford, CA, USA) and Java TreeView 3.0 (Stanford University, USA). The differential abundance of metabolites was analyzed with deseq2 [47]. Metabolic pathway enrichment analysis was carried out using the R package FELLA [48] with the reference data downloaded from KEGG (organism: mtu). The volcano plot was generated by R package ggplot2 [49] and ggrepel (https://CRAN.R-project.org/package=ggrepel) (accessed on 3 November 2021). The network plot of pathways and metabolites was generated by Cytoscape [50].

### 4.11. Drug Susceptibility Analysis

For the drug susceptibility assay, biofilms of *M. tuberculosis* were grown in 96-well polystyrene plates, each well containing 100 μL of serum-7H9 media containing a saturated planktonic culture in the presence or absence of 2 μM of Epi. The plates were parafilm-wrapped and incubated at 37 °C for 1 week. Epi-induced M. tuberculosis biofilms were treated with RIF (MIC = 0.025 μg/mL), INH (MIC = 0.025 μg/mL), and Bedaquiline (MIC = 0.02 μg/mL) at 0×, 1×, 5×, and 10× MIC, respectively, and then incubated for 48 h at 37 °C. Following incubation, 20 μL of 0.02% Resazurin (sodium salt, MP Biomedicals) was added to each of the wells of the plates and color change was monitored after incubation of approximately 20 h at 37 °C. Experiments were performed independently three times, each with triplicate determinations.

### 4.12. In Vitro Stress Susceptibility Assay

To investigate the effect of Epi on the different stress responses of *M. tuberculosis*, biofilms of *M. tuberculosis* were grown in 96-well polystyrene plates, each well containing 100 μL of serum-7H9 media in the presence or absence of 2 μM of Epi. The plates were parafilm-wrapped and incubated at 37 °C for 1 week. Epi-induced *M. tuberculosis* biofilms were treated with 0.1% SDS, 10 mM H_2_O_2_, low pH (pH 4.5, adjusted with HCl), and 5% ethanol, respectively, and then incubated for 48 h at 37 °C. Following incubation, 20 μL of 0.02% Resazurin (sodium salt, MP Biomedicals, Santa Ana, CA, USA, 0219459801) was added to each of the wells of the plates and color change was monitored after incubation of approximately 20 h at 37 °C. Experiments were performed independently three times, each with triplicate determinations.

### 4.13. Other Insilco Analysis

The *M. tuberculosis* target genes and proteins were identified using the online database mycobrowser available at https://mycobrowser.epfl.ch/ (accessed on 6 March 2019).

### 4.14. Statistical Analysis

Numerical data were analyzed and plotted by using GraphPad Prism 7.0 (La Jolla, CA, USA) software from three independent experiments shown as mean ± SD or SEM. Evaluation of the significance of differences between groups was performed by using two-way ANOVA or Student’s *t* test. Statistical differences were considered significant when *p* < 0.05 and the *p* values of <0.05, <0.01, <0.001, and <0.0001 were indicated as *, **, *** and **** in figures, respectively.

## 5. Conclusions

Our data revealed that epinephrine stimulates *M. tuberculosis* growth and biofilm formation, which contributes to the understanding of the environmental perception and antibiotic resistance of *M. tuberculosis*.

## Data Availability

The raw and processed data of metabolome are available at https://ngdc.cncb.ac.cn/omix under the accession number OMIX003595.

## Supplementary Materials

The supporting information can be downloaded at https://www.mdpi.com/article/10.3390/ijms242417370/s1.
