# Supplementary material for: Epinephrine Stimulates Mycobacterium tuberculosis Growth and Biofilm Formation
Source: Int J Mol Sci. 2023 Dec 12;24(24):17370. doi: 10.3390/ijms242417370 (PMC10743465; doi:10.3390/ijms242417370)
Supplement: Supplementary file 1 [file ijms-24-17370-s001.zip › ijms-2696494-supplementary figures.pdf]

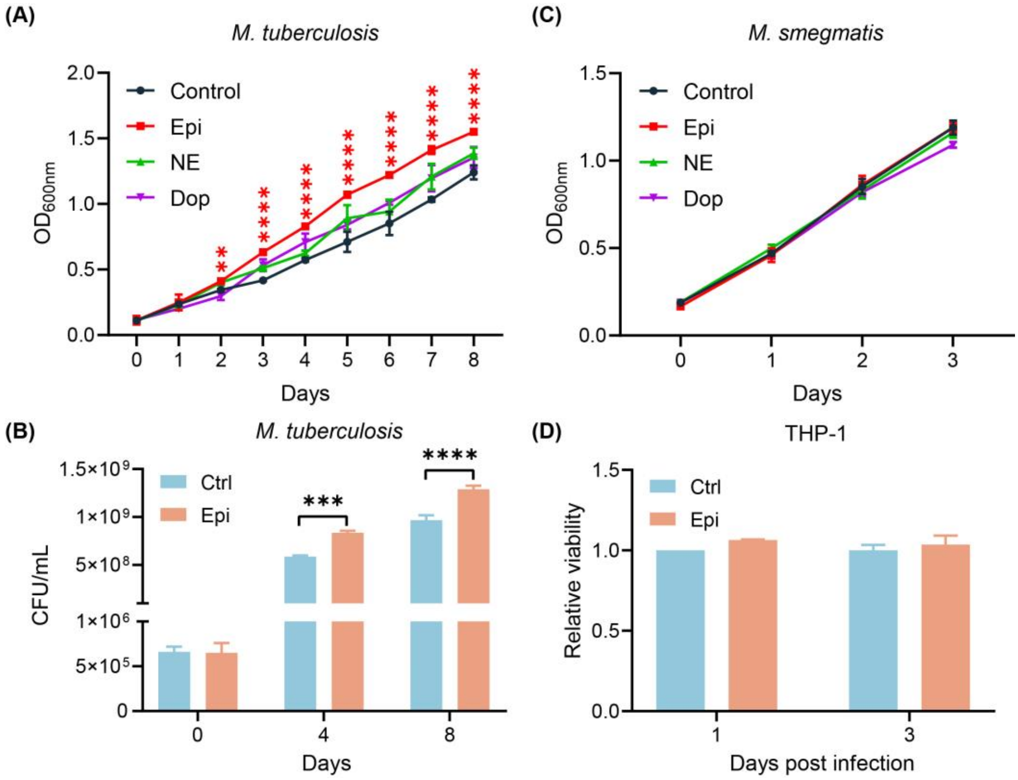


**Figure S1.** Effects of catecholamine stress hormones on *M. tuberculosis* and *M. smegmatis*. (A) Effects of catecholamine stress hormones (2 μM Epi, 2 μM NE, and 2 μM Dop) on the growth of *M. smegmatis* on serum-7H9 medium. (B) Effects of Epi on *M. tuberculosis* CFU *in vitro*. (C) Effects of catecholamine stress hormones (2 μM Epi, 2 μM NE, and 2 μM Dop) on the growth of *M. smegmatis* on serum-7H9 medium. (D) THP-1 cell viability was assessed by MTS assay. ****, $p < 0.0001$; ***, $p < 0.001$; **, $p < 0.01$ (two-way ANOVA). Data are representative of three independent experiments with three biological replicates (mean ± SEM).

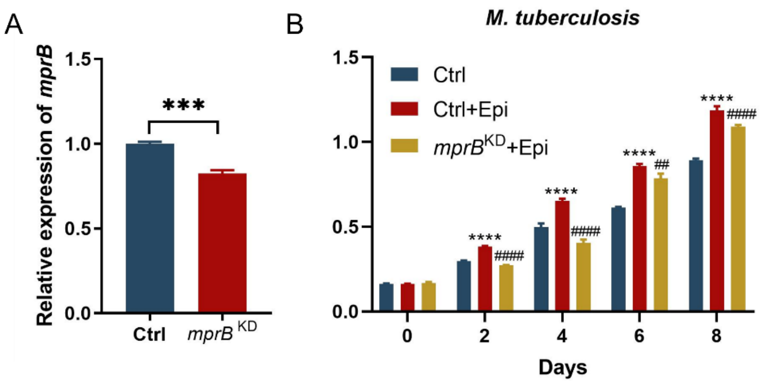


**Figure S2.** MprB is involved in regulation of *M. tuberculosis* growth *in vitro* by Epi. (A) Confirmation of *mprB* knocked down in *M. tuberculosis* by qRT-PCR. The data are represented as mean ± SEM. Statistical significance was determined using Student's *t* test. ***, $p < 0.001$. (B) Effects of Epi on *mprB* knocked down *M. tuberculosis* strain as compared to the wild-type strain. **** and ####, $p < 0.0001$; ##, $p < 0.01$ (two-way ANOVA). Data are representative of three independent experiments with three biological replicates (mean ± SD).
